# Effects of the In Situ Growth of CNTs on Ti-Coated Diamond Surfaces on the Mechanical Properties of Diamond/Aluminum Composites

**DOI:** 10.3390/nano14070640

**Published:** 2024-04-07

**Authors:** Hao Wu, Ping Zhu, Yixiao Xia, Yifu Ma, Junyao Ding, Huasong Gou, Qiang Zhang, Sen Yang, Gaohui Wu

**Affiliations:** 1 School of Materials Science and Engineering, Nanjing University of Science and Technology, Nanjing 210094, China; wh@njust.edu.cn; 2 School of Materials Science and Engineering, Harbin Institute of Technology, Harbin 150001, China; 18846450834@163.com (P.Z.); etialxia@icloud.com (Y.X.); tyl563@163.com (Y.M.); dingjunyaoheu@163.com (J.D.); ghs@hit.edu.cn (H.G.); wugh@hit.edu.cn (G.W.); 3 Key Laboratory of Advanced Structure-Function Integrated Materials and Green Manufacturing Technology, School of Material Science and Engineering, Harbin Institute of Technology, Harbin 150001, China

**Keywords:** diamond/aluminum composites, CNT, interface reaction, bending strength, interface microstructure

## Abstract

Diamond/aluminum composites have attracted significant attention as novel thermal management materials, with their interfacial bonding state and configuration playing a crucial role in determining their thermal conductivity and mechanical properties. The present work aims to evaluate the bending strength and thermal conductivity of CNT-modified Ti-coated diamond/aluminum composites with multi-scale structures. The Fe catalyst was encapsulated on the surface of Ti-coated diamond particles using the solution impregnation method, and CNTs were grown in situ on the surface of Ti-coated diamond particles using the plasma-enhanced chemical vapor deposition (PECVD) method. We investigated the influence of interface structure on the thermal conductivity and mechanical properties of diamond/aluminum composites. The results show that the CNT-modified Ti-coated diamond/aluminum composite exhibits excellent bending strength, reaching up to 281 MPa, compared to uncoated diamond/aluminum composites and Ti-coated diamond/aluminum composites. The selective bonding between diamond and aluminum was improved by the interfacial reaction between Ti and diamond particles, as well as between CNT and Al. This led to the enhanced mechanical properties of Ti-coated diamond/aluminum composites while maintaining acceptable thermal conductivity. This work provides insights into the interface’s configuration design and the performance optimization of diamond/metal composites for thermal management.

## 1. Introduction

With the continuous miniaturization and integration of lightweight modern electronic equipment, the demand for advanced thermal management materials is increasing [1,2,3]. Diamond/aluminum composites, regarded for their unique properties such as high thermal conductivity [4,5,6] and an adjustable coefficient of thermal expansion [7,8], have found applications in thermal management materials for electronic packaging. The diamond/aluminum interface, as a nonmetal/metal interface, is highly non-wetting and acoustically mismatched [9,10], so the performance of the composite is determined by the interface between diamond and aluminum. Additionally, Al_4_C_3_, a brittle and easily hydrolyzed interfacial product, is prone to be formed in the preparation process of diamond/aluminum composites [11,12,13,14]. The interfacial reaction promotes the interfacial bonding between diamond and aluminum, allowing for the fabrication of diamond/aluminum composites with high thermal conductivity by controlling the content and size of Al_4_C_3_ [10,15]. However, the requirements of thermal management materials extend beyond high thermal conductivity and good interface bonding, the high reliability of the composites is also essential. It is reported that when diamond/aluminum composites were immersed in water at room temperature for 115 days, the thermal conductivity of composites decreased from 467 W/(m·K) to 347 W/(m·K), and the tensile strength decreased from 105 MPa to 61 MPa [16].

Currently, researchers have developed numerous methods to optimize the diamond/aluminum interface to address the issues mentioned above, such as introducing an interface layer that bridges acoustic mismatch and changing the surface state of diamond particles [12,17,18,19,20,21,22,23,24,25]. Xue et al. [26] modified the interface of diamond/aluminum composites by coating Ti on the surface of diamond particles and adding Ti to the matrix. It was found that when the nominal titanium content was the same, the thermal conductivity of Ti-coated diamond/aluminum composites was higher than that of diamond/Ti-aluminum composites, with the highest reaching 475 W/(m·K). Dong et al. [27] inserted a 20 nm SiC interface layer at the interface between diamond and aluminum, resulting in a thermal conductivity of 485 W/(m·K) for the composite. Compared with the uncoated diamond/aluminum composite, the thermal conductivity of a ZrC-coated diamond/aluminum composite prepared by Li et al. [28] was reduced from 709 W/(m·K) to 572 W/(m·K), which inhibited the formation of Al_4_C_3_, a harmful interface product prone to hydrolysis, while maintaining acceptable thermal conductivity. In addition, Monachon et al. [20] also reported the effect of diamond terminals on the thermal conductivity of the diamond/aluminum interface. Their experimental results indicate that, compared to the H-terminal diamond/aluminum interface, the oxygen-terminal diamond/aluminum interface displays higher interfacial thermal conductance. While this study provides a new perspective for exploring interface modification [20], it did not consider the influence of C-Al reactions on the thermal conductivity of composites.

For diamond/metal composites, interfacial in situ growth has also been applied to enhance the thermal conductivity of composites. Xu et al. developed a diamond/copper composite with a thermal conductivity as high as 711 W/(m·K) by employing a scalable monolayer-functionalized (SAM) nanointerface, where the diamond particle size and volume fraction were 200 μm and 50%, respectively [29]. The in situ growth of graphene between diamond and copper has also been attempted to improve the wettability and alleviate acoustic mismatch between diamond and copper [30]. Experiments have demonstrated that the introduction of graphene interlayers plays a positive role. However, research on in situ growth for the interfacial modification of diamond/aluminum composites is rarely reported.

For diamond/aluminum composites, more research focuses on their thermal conductivity. The mechanical properties, friction properties, and optical properties of diamond/aluminum composites, which have attracted the interest of some researchers, are less reported. Wu et al. [31] found that when the coating time of Ti on a diamond surface via the molten salt method was 90 min, the corresponding diamond/aluminum composite obtained a high tensile strength of 76 MPa. Chen et al. [32] showed that the wear resistance of diamond/aluminum composites prepared via solid-state cold spray additive manufacturing technology is comparable to that of selective laser-formed Inconel625 and 17−4PH alloys. Bellucci et al. [33] reported the photoelectric application of nanostructured diamond/aluminum composites with visible radiation spectra.

In this study, we synthesized CNTs grown in situ on the surface of Ti-coated diamond particles and investigated the bending strength of CNT-modified Ti-coated diamond/aluminum composites with multi-scale structures. The aim of this study is to elucidate the interfacial structure of diamond/aluminum composites and clarify the influence of multi-scale nanomodification structures on the performance of composite.

## 2. Materials and Methods

### 2.1. Materials

As the matrix material, 1060 bulk aluminum with a purity of 99.99 wt.% was purchased from Northeast Light Alloy Co., Ltd., Harbin, China. Monocrystalline diamond particles (MBD4 type, Henan Huanghe Whirlwind Co., Changge, China) with an average particle size of 355 μm were used as reinforcements for the composites. The surface of a diamond is composed of six square {100} planes and eight hexagonal {111} planes.

### 2.2. Preparation of CNT−Ti−Diamond Multiscale Architectures

The Ti coating on the diamond particles was prepared via magnetron sputtering, with a thickness of 300 nm. Then, Fe(NO_3_)_3_·9H_2_O acted as a catalyst to coat the Ti-coated diamond surface. First, the Ti-coated diamond was immersed in a 0.05 mol/L Fe(NO_3_)_3_·9H_2_O solution, stirred for 4 h using a magnetic stirrer, and then soaked at room temperature for 12 h. The solution was poured off, and the diamond particles coated with Fe(NO_3_)_3_·9H_2_O were dried. Ti-coated diamond particles loaded with Fe(NO_3_)_3_·9H_2_O were moved into a PECVD reaction chamber. Methane was used as a carbon source, and hydrogen was used as a reducing gas and carrier gas. Ti-coated diamond particles coated with Fe(NO_3_)_3_·9H_2_O were heated to 650 °C at a rate of 10 °C/min and held for 30 min under the protection of H_2_ with a flow rate of 20 sccm, and then Fe^3+^ was reduced to metallic Fe. Then, the radio frequency (RF) was set at 13.56 MHz to generate plasma for 20 min, and carbon nanotubes were grown on the surface of diamond particles after the methane/hydrogen mixture was introduced into the CVD reactor at a flow rate of 20/5 sccm at a total pressure of 140~160 Pa. After the reaction was completed, the RF and heating power supply were turned off. The reactor was cooled to room temperature in a H_2_ atmosphere. Finally, the multi-scale structure of CNT−Ti−diamond was prepared and taken out.

### 2.3. Fabrication of Diamond/Al Composite

The CNT-modified Ti-coated diamond/Al composites with a diamond volume fraction of 60% were prepared using the gas-assisted pressure infiltration (GPI) method. The details are as follows: First, diamond particles were filled into a graphite mold and vibrated. Afterward, the aluminum ingot was placed on the top of the mold containing diamond particles, and they were placed in the furnace together. Subsequently, the mold with diamond was preformed, and an aluminum ingot was then heated to 800 °C at a heating rate of 30 °C/min, where it stayed for 20 min under vacuum; then, it was pressurized until the gas pressure reached 15 MPa to complete the infiltration process. Finally, it was cooled down to room temperature in a furnace, and diamond/aluminum composites were obtained. Uncoated diamond/Al composites and untreated Ti-coated diamond/aluminum composites were fabricated via the same process for comparison.

### 2.4. Characterization

X-ray diffraction (XRD, Philips Company, Almelo, Netherlands) with Cu-Kα radiation was used to analyze the phase structure of diamond particles and diamond/Al composites. A Quanta 200FEG (Hillsboro, OR, USA) field emission environmental scanning electron microscope (SEM) was employed to characterize the microstructure of the diamond particles, as well as the fracture morphology of diamond/Al composites.

Three-point bending tests were conducted on an Instron 5569 universal electrical tensile testing machine (Instron, Boston, MA, USA) with a span of 30 mm and a crosshead speed of 0.5 mm/min, with rectangular samples of 3 mm × 4 mm × 36 mm. Three samples of each type of composite were tested to avoid the influence of accidental errors. All samples were polished with sandpaper before testing. The thermal conductivity of the obtained diamond/Al composites was derived from the equation *λ* = *αρc*, where *λ*, *α*, *ρ*, and *c* represent thermal conductivity, thermal diffusivity, the density of the composite, and specific heat capacity, respectively. Thermal diffusivity (*α*) was measured using the LFA 467 Nanoflash instrument(Netzsch, GmbH, Selb, Germany) at room temperature with disk-shaped samples of *Φ*12.7 mm × 3 mm. The specific heat capacity was determined by the rule of mixture (ROM).

## 3. Results

### 3.1. Morphology of Modified Diamond Particles

**Figure 1:**
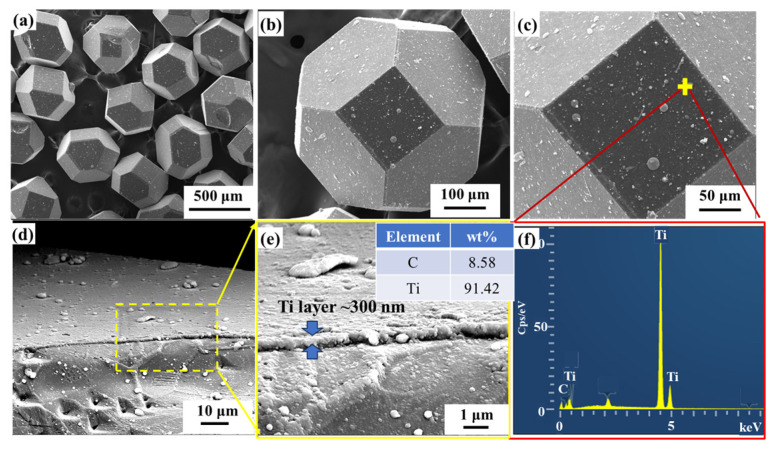
Morphology of untreated Ti-coated diamond particles: (**a**) SEM image; (**b**,**c**) magnified SEM image; (**d**) SEM of the broken diamond; (**e**) SEM and EDS spectrum analysis of broken diamond coating in the yellow box of (**d**); (**f**) EDS spectrum at the mark in (**c**).

The morphology of obtained Ti-coated diamond particles is shown in Figure 1. It can be observed that the diamond exhibits a regular shape, and massive bright spherical substances are distributed on the surface of the diamond. Both the quadrilateral {100} and hexagonal {111} crystal planes of the diamond particles are coated with a Ti layer. EDS spectrum analyses of the micro-region containing the spherical substances show two elements, Ti and C. The low magnetron sputtering temperature is not sufficient to make the diamond react with Ti, indicating that the spherical material is titanium, and this is caused by the solidification and aggregation of Ti in the process of magnetron sputtering. The Ti-coated diamond particles were ball-milled to break the Ti coatings, and then the thickness of the Ti coatings was determined to be about 300 nm according to Figure 1e.

**Figure 2:**
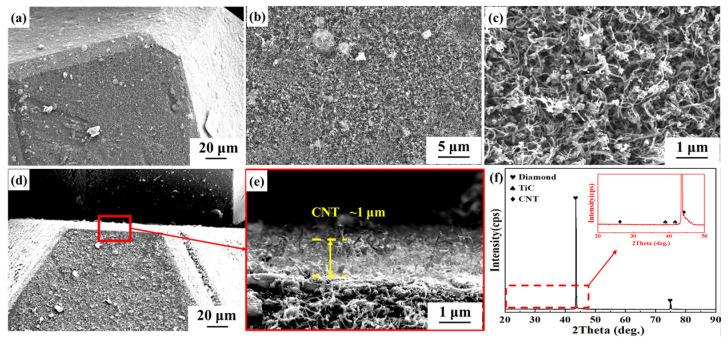
Surface morphology and phase analysis of Ti-coated diamond particles modified by CNT: (**a**,**d**) SEM image; (**b**,**c**,) morphology of CNT on the surface of Ti-coated diamond; (**e**) Enlarged image at the red box in (**d**); (**f**) XRD patterns of CNT-modified Ti-coated diamond particles.

CNT-modified Ti-coated multi-scale modified diamond reinforcements were prepared to enhance the mechanical properties of Ti-coated diamond/aluminum composites. Figure 2 shows the microstructure and phase analysis of in situ CNT growth on the surface of Ti-coated diamond particles by PECVD. It can be seen that CNTs are formed on all crystal faces of the diamond particles. From the enlarged Figure 2b, it can be seen that high-density CNTs are distributed on the diamond surface, and some CNTs exhibit bending and entanglement phenomena.

The thickness of CNT generated in situ on the surface of Ti-coated diamond is approximately 1 μm (Figure 2e). Based on the thickness of Ti coatings on the diamond surface and the observed length of CNT, we estimated the volume fractions of CNT, Ti, and diamond in CNT-modified Ti-coated diamond to be 1.53%, 0.45%, and 98.02%, respectively. For CNT-modified Ti-coated diamond particles, the XRD pattern in Figure 2f reveals extremely strong diamond diffraction peaks. While CNTs were also detected, their diffraction peaks were weak due to their low content. Fe(NO_3_)_3_, serving as the precursor of the catalyst, was reduced to Fe to realize the growth of CNT on the surface of Ti-coated diamonds. Previous reports suggest that the growth of CNT on the Ti surface via the CVD method follows the top growth mode, the catalytic particles are coated by C atoms in the CNT, and finally, the CNT is precipitated from the bottom of the catalyst [34]. Consequently, some Fe remains, and the grown CNT accounts for 1.53 vol.% of the CNT-modified Ti-coated diamond and 0.92 vol% of the CNT-modified Ti-coated diamond/aluminum composite. As a result, the corresponding Fe content is even lower, making it difficult to detect.

### 3.2. Microstructure of Diamond/Al Composites

**Figure 3:**
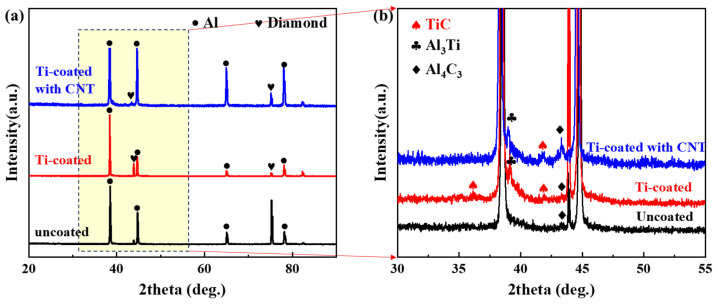
(**a**) XRD pattern of diamond/aluminum composite; (**b**) enlarged pattern of the interval corresponding to the box in (**a**).

CNT-modified Ti-coated diamond/aluminum composites were prepared via gas-assisted pressure infiltration, and Ti-coated and uncoated diamond/aluminum composites were also fabricated using the same process for comparison (denoted as CNT-modified Ti-coated diamond/Al composites, Ti-coated diamond/Al composites, and uncoated diamond/Al composites, respectively). Figure 3 shows the XRD patterns of the above three diamond/aluminum composites. In addition to the basic Al and diamond diffraction peaks (shown in Figure 3a), it can be seen in Figure 3b that TiC and Al_3_Ti are generated in CNT-modified Ti-coated diamond/aluminum composites and Ti-coated diamond/aluminum composites. The TiC diffraction peak of the CNT-modified Ti-coated diamond/aluminum composite shows a higher peak intensity, which means that the higher content of TiC is attributed to the extra formation of TiC in the process of in situ growth of CNTs. In addition, the diffraction peak of Al_4_C_3_ was detected in the uncoated diamond/aluminum composite and the CNT-modified Ti-coated diamond/aluminum composite, and the generation of Al_4_C_3_ was attributed to different reactions. In uncoated diamond/aluminum composites, Al_4_C_3_ is formed via the interfacial reaction of molten aluminum with diamonds. However, for CNT-modified Ti-coated diamond/aluminum composites, Al_4_C_3_ is formed via the reaction of molten aluminum with CNTs formed in situ on the surface of Ti-coated diamond.

**Figure 4:**
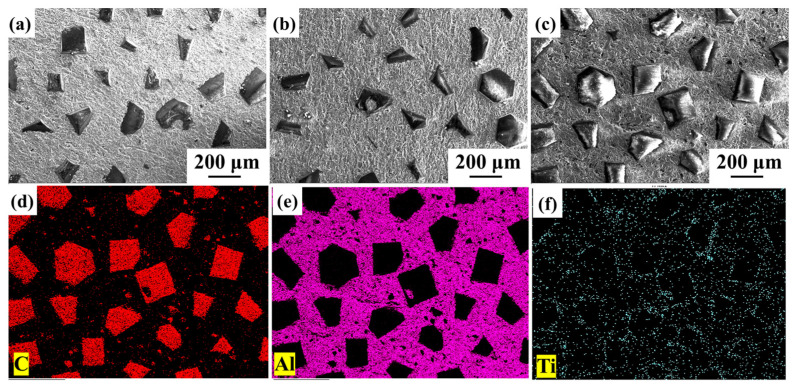
Surface morphology of diamond/aluminum composite: (**a**) uncoated diamond/aluminum composite; (**b**) Ti-coated diamond/aluminum composite; (**c**) CNT-modified Ti-coated diamond/aluminum composite; (**d**–**f**) EDS mapping analysis of (**c**).

Figure 4 shows the microstructure of the prepared diamond/aluminum composite. It can be seen in Figure 4a–c that the diamond is uniformly distributed in the aluminum matrix, which is similar to the results reported in Refs. [15,35]. Since the diamond is hexoctahedral rather than spherical, only a small part of the diamond particles is exposed to the surface. According to the EDS mapping analysis, Ti elements are aggregated at the interface of CNT-modified Ti-coated diamond/aluminum composites.

**Figure 5:**
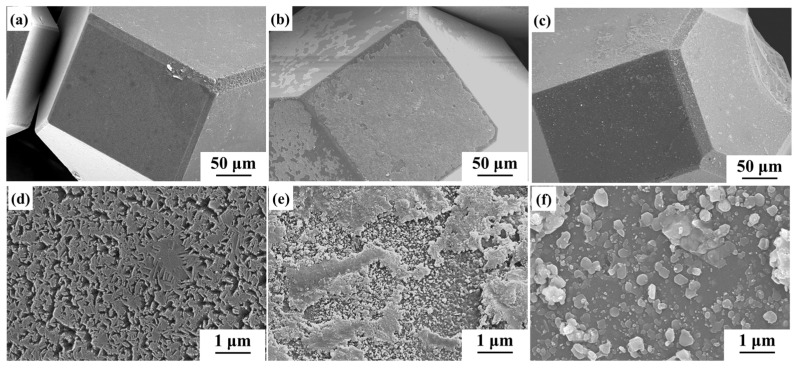
Surface morphology of diamond particles extracted from diamond/aluminum composites: (**a**,**d**) uncoated diamond/aluminum composite; (**b**,**e**) Ti-coated diamond/aluminum composite; (**c**,**f**) CNT-modified Ti-coated diamond/aluminum composite.

Then, hydrochloric acid is used to corrode the Al matrix in the diamond/aluminum composite, thereby extracting the diamond from the composite for further analysis. Figure 5 depicts the surface morphology of diamond particles extracted from several diamond/aluminum composites, showing significant differences.

The surface of diamond particles extracted from uncoated diamond/aluminum composites shows a large number of conical corrosion pits. A similar pyramid-shaped corrosion pit was also observed in Kleiner’s research on diamond/aluminum composites [36], and combined with TEM, it was confirmed that the corrosion pit originated from the formation of Al_4_C_3_ at the interface. Due to the hydrolysis of Al_4_C_3_ during the corrosion process, no Al_4_C_3_ remains on the surface of the extracted diamond particles, and according to the morphology of the diamond surface, it can be seen that Al_4_C_3_ is generated by the contact reaction between molten aluminum and diamond and grows in the direction embedding inside the diamond. A jagged structure is formed at the interface between diamond and aluminum, which enhances the interfacial bonding by increasing the contact area between diamond and aluminum and the bridging effect of Al_4_C_3_ on diamond and aluminum.

The retained interfacial products were observed on the surface of diamond particles extracted from untreated Ti-coated diamond/aluminum composites. Combined with the XRD analysis of Figure 3, it was confirmed that the discontinuous layered phase distributed on the surface of diamond particles was TiC. However, the diamond surface extracted from the CNT-modified Ti-coated diamond/aluminum composite was completely covered by layered interface products, and no exposed diamond surface was observed, indicating that the TiC formed in this condition is more complete and uniform.

### 3.3. Mechanical and Thermal Properties

Table 1 shows the bending strength and thermal conductivity of three kinds of diamond/aluminum composites. The in situ formation of CNTs on the surface of Ti-coated diamond particles improves the bending strength of the composite. As a thermal management material, thermal conductivity is also an important performance indicator. Therefore, we also evaluated the thermal conductivity of diamond/aluminum composites. Among the three kinds of composites, uncoated diamond/aluminum composites show the best thermal conductivity. With the introduction of the Ti coating, the thermal conductivity and bending strength of composites were obviously reduced. The CNT-modified Ti-coated diamond/aluminum composite possesses the highest bending strength despite sacrificing part of the thermal conductivity.

**Figure 6:**
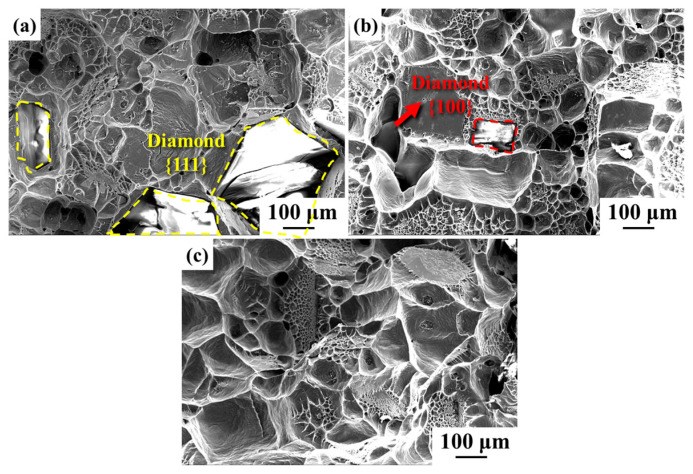
Fracture morphology of the diamond/aluminum composite: (**a**) uncoated diamond/aluminum composite; (**b**) Ti-coated diamond/aluminum composite; (**c**) CNT-modified Ti-coated diamond/aluminum composite.

Figure 6 illustrates the fracture morphology of the diamond/aluminum composite. The phenomenon of the selective combination of different planes of diamond with aluminum appears in the uncoated diamond/aluminum composite, as shown in Figure 6a. The diamond {111} plane is exposed and debonded from the aluminum matrix, while the diamond {100} plane adheres to the aluminum. This is attributed to the difference in interface properties between diamond {100} and {111} planes and aluminum. The interface bonding between diamond {100} crystal planes and aluminum is stronger, and it also has a stronger tendency to form Al-C bonds [37].

The pulling-out phenomenon of diamond particles can be observed in the fracture structure of Ti-coated diamond/aluminum composites. The diamond {111} plane is well bonded with aluminum, while the interface bonding with aluminum on the diamond {100} plane is either interfacial debonding or broken between the interface layer and aluminum. After the in situ growth of CNTs on the surface of Ti-coated diamond particles, the fracture surface of the resulting composite showed excellent interfacial bonding. All diamond planes adhered to aluminum, and the selective bonding of different diamond planes with aluminum was improved.

**Figure 7:**
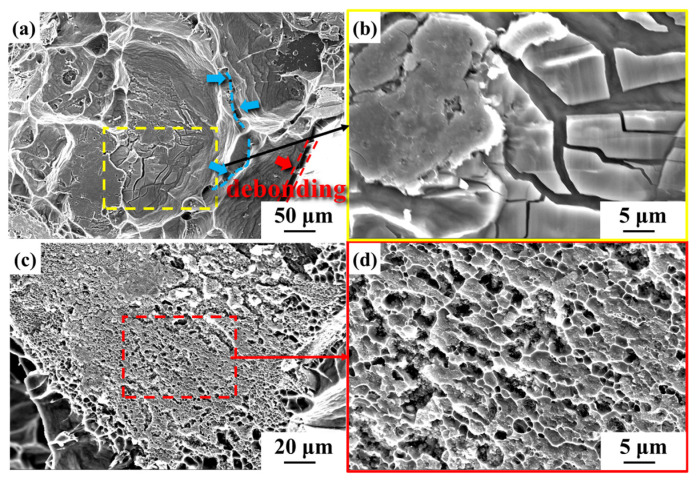
Fracture morphology of uncoated diamond/aluminum composite (**a**,**c**) and corresponding enlarged images (**b**,**d**).

The detailed characterization of the fracture morphology of uncoated diamond/aluminum composite is shown in Figure 7. As can be seen from the red arrow in Figure 7a, there is an area of diamond and aluminum interface debonding at the uncoated diamond/aluminum interface. Although no obvious fracture was observed, discontinuous cracks were located at the interface between some diamond edges and aluminum, as shown by the blue dotted line indicated by the blue arrow. Figure 7b shows that the brittle phase exists at the fracture area of diamond and aluminum, and the brittle phase breaks during the bending strength test. The formation of brittle phase Al_4_C_3_ transforms the mechanical bonding between diamond and aluminum into chemical bonding so that the fracture surface of the composite also shows a certain dimple morphology.

**Figure 8:**
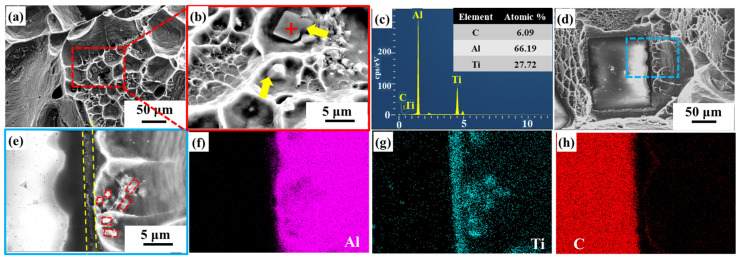
Fracture surface and composition analysis: (**a**,**d**) fracture morphology of Ti-coated diamond/aluminum composite; (**b**) morphology of precipitated phases in aluminum matrix in the red box of (**a**); (**c**) EDS analysis result of the phase marked by a red cross in (**b**); (**e**) interface of diamond and aluminum marked by a blue box in (**d**); (**f**–**h**) EDS element distribution mapping of Al, Ti, and C in (**e**), respectively.

For Ti-coated diamond/aluminum composites, as shown in Figure 8a, massive dimples can be observed, with block-like precipitates distributed in the dimples. EDS spectroscopy analysis was performed on the blocky phase in Figure 8b, and the results showed that it was mainly composed of Al and Ti elements, with an atomic ratio of approximately 3:1. Combined with XRD analysis, it was confirmed that the precipitated phase was the intermetallic compound Al_3_Ti, attributed to the reaction between molten aluminum and Ti during the infiltration process. Meanwhile, an interface layer existed at the interface between diamond and aluminum, as shown in Figure 8e. The EDS mapping analysis of composites surface in Figure 8e shows that Ti elements gather at the interface Combined with XRD, the interface layer is confirmed as TiC generated by the reaction between diamond and Ti. Additionally, Al and Ti elements are also clustered in the red box in Figure 8e, and they are identified as Al_3_Ti.

Therefore, in the untreated Ti-diamond/aluminum composite, on the one hand, Ti reacts with diamond to form TiC. On the other hand, the TiC layer acts as the intermediate layer between Ti and C, slowing down the diffusion reaction between the two. At this time, Ti, which is in contact with molten aluminum, preferentially reacts with it to form Al_3_Ti, resulting in the interface structure depicted in Figure 8. In the Ti-coated diamond/aluminum composite, the dominant interface structure is diamond/TiC/Al. For diamond {111}/TiC/Al, the fracture occurs at the aluminum matrix, with numerous dimple morphologies, indicating a significant improvement in bonding between the diamond {111} plane and aluminum. Interestingly, for diamond {100}/TiC/Al, more fractures occur between TiC and aluminum, which may be related to the difference in the reaction rates between different crystal planes of diamond and Ti. Generally, the more active diamond {100} plane tends to have a higher reaction rate with Ti, which will be investigated in our future work.

**Figure 9:**
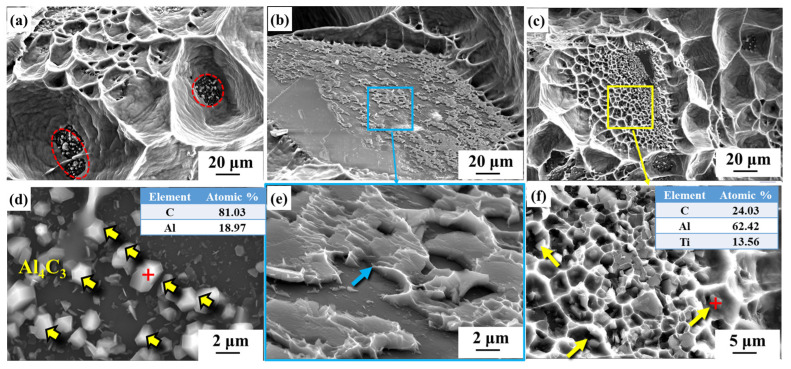
Fracture morphology of CNT-modified Ti-coated diamond/aluminum composites (**a**–**c**) and corresponding enlarged images (**d**–**f**).

In the CNT-modified Ti-coated diamond/aluminum composite shown in Figure 9, a flaky precipitated phase was also observed to be distributed in the Al matrix, with a smaller size than that generated in the Ti-coated diamond/aluminum composite (Figure 9f). According to the analysis of EDS elements, the marked position contains Al, C, and Ti, which is similar to that in Figure 8b, and combined with XRD, it is considered Al_3_Ti. During the in situ formation of CNTs via the surface treatment of Ti-coated diamonds, diamond particles reacted with Ti to form TiC. In the subsequent infiltration process, TiC continued to form, so the amount of content of residual Ti reacting with molten Al decreased, and the size and quantity of the Al_3_Ti formed were also reduced. In addition, in Figure 9a, a large number of polyhedral interface products appeared at the position marked by a red cross, where elemental analysis showed only two elements, Al and C. It was attributed to the reaction between molten aluminum and CNT during the infiltration process. Meanwhile, the phenomenon of Al adhering to the {100} surface of the diamond was also observed, suggesting that the generated Al_4_C_3_ played a certain role in bonding diamond and aluminum.

**Figure 10:**
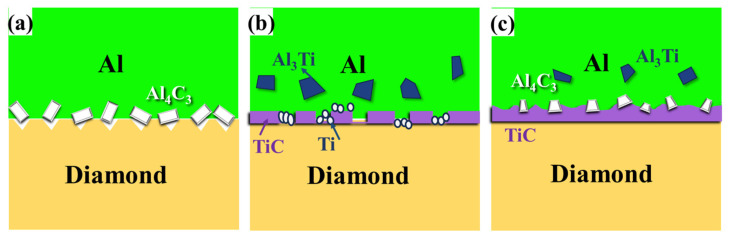
Schematic diagram of the interface structure of diamond/aluminum composites: (**a**) uncoated diamond/aluminum composite; (**b**) Ti-coated diamond/aluminum composite; (**c**) CNT modified Ti-coated diamond/aluminum composite.

Figure 10 depicts the schematic diagram of the interface structure of different diamond/aluminum composites. The Al-C interface reaction occurs at the interface of the uncoated diamond/aluminum composite. According to Figure 5d, the interface product Al_4_C_3_ is embedded into the diamond particle’s surface, showing a zigzag distribution. The interface is closely connected through the interface reaction between diamond and aluminum, forming Al_4_C_3_. A discontinuous interface layer TiC is formed at the interface of the Ti-coated diamond/aluminum composite, with large-sized Al_3_Ti also distributed in the aluminum matrix. Since Al_3_Ti is a brittle phase, its smaller size can bear loads and hinder dislocation movement; however, excessively large sizes can bring adverse effects. TiC is generated in situ from diamond and Ti, so compared with TiC and Al, the TiC/diamond interface bonding is stronger, and some TiC and aluminum interface debonding occurs in the fracture. For CNT-modified Ti-coated diamond/aluminum composites, on the one hand, Ti reacts with diamond to form TiC; on the other hand, the pre-formed CNT reacts with aluminum to form a small amount of Al_4_C_3_, and diamond and aluminum are bridged by various products, which enhances the bending strength of Ti-coated diamond/aluminum composites and improves the selective bonding between diamond and aluminum.

The thermal conductivity of composites is controlled by the synergistic effect of the interface bonding strength and interface structure. Generally, the higher the interfacial bonding strength of composites, the better the thermal conductivity of the corresponding composites. When the interface is well combined, the interface structure will play a leading role in the thermal conductivity of the composite, which is mainly related to the composition (acoustic performance matching) and content of the interface phase.

In this study, according to the results, it is observed that the vibration matching of interfacial phonons is more influential than interfacial bonding. This is attributed to the fact that although the bending strength of diamond/aluminum composites shows differences, the composites produced by the gas pressure infiltration method do not present obvious defects and voids, while noticeable differences are observed in the interface structure of the composites. For uncoated diamond/aluminum composites, Al reacts with C at the interface to form Al_4_C_3_. Indeed, high-thermal-conductivity diamond/aluminum composites can be achieved through the formation of an appropriate amount of Al_4_C_3_. Monje et al. [10] improved the thermal conductivity of diamond/aluminum materials from 390 W/(m·K) to 670 W/(m·K) by adjusting the preparation process of gas pressure infiltration. However, the author did not evaluate the difference in the bending strength of the composites.

The introduction of the Ti interface modification layer can suppress the generation of Al_4_C_3_, but it also increases the number of interfaces and introduces additional interface thermal resistance. The final interface composition in the Ti-coated diamond/aluminum composite is TiC and large-sized Al_3_Ti (up to 5 μm). The formation of Al_3_Ti in the Al matrix results in extra phonon scattering, leading to a decrease in the thermal conductivity of Ti-coated diamond/aluminum composites compared with uncoated diamond/aluminum composites. A similar phenomenon has also been reported in Ref. [24]. Ma et al. inserted Mo_2_C into the diamond/aluminum interface, resulting in a decrease in the thermal conductivity of the composites from 553 W/(m·K) to 218 W/(m·K) [24]. The authors attributed the decrease in thermal conductivity to the intermetallic compound Al_12_Mo.

As for the CNT-modified Ti-coated diamond/aluminum composite, although the bending strength of the composite is improved through the multi-interface reaction between diamond and Ti, CNTs, and Al, some thermal conductivity is also sacrificed. First, compared with Ti-coated diamond/aluminum composites, CNT-modified Ti-coated diamond/aluminum composites produce more TiC, corresponding to the stronger TiC diffraction peak in Figure 3a. Theoretical calculations carried out by Tan et al. show that an increase in coating thickness will lead to a decrease in the thermal conductivity of diamond/aluminum composites [38]. In addition, in the CNT-modified Ti-coated diamond/aluminum composite, CNT reacts with aluminum during infiltration to produce Al_4_C_3_, which makes the composite exhibit the interfacial structure of diamond/TiC/Al_4_C_3_/Al, further increasing the interface’s thermal resistance, so thermal conductivity is the lowest.

## 4. Conclusions

Uncoated, Ti-coated, and CNT-modified Ti-coated diamond/aluminum composites were prepared using the gas pressure infiltration method. The interface structure of the composites was characterized, and the mechanical properties and thermal conductivity of the composites were investigated. The main conclusions are summarized as follows:

(1) CNTs have been successfully grown on the surface of Ti-coated diamond particles using the PECVD method, with a length of approximately 1 μm.
(2) The CNT-modified Ti-coated diamond/aluminum composite achieves an increase in the bending strength through the interfacial reaction during the infiltration process, which makes the diamond and aluminum closely connected through TiC and Al_4_C_3_. The bending strength of the CNT-modified Ti-coated diamond aluminum composite is increased by about 9% compared with the uncoated diamond/aluminum composite.
(3) The thermal conductivity of composites is not only contributed by interface bonding, but it is also closely related to the interfacial structure. The thermal conductivity of Ti-coated diamond/aluminum composites and CNT-modified Ti-coated diamond/aluminum composites is lower than that of uncoated diamond/aluminum composites, and this is attributed to the formation of large-sized Al_3_Ti and the generation of thicker interface phases, respectively.
(4) CNT-modified Ti-coated diamond/aluminum composites achieve a balance between improved mechanical properties and acceptable thermal conductivity. This study provides a promising strategy for the design and preparation of high-performance diamond/metal using the interface configuration design method.

## Figures and Tables

**Table 1:** Performance of diamond/aluminum composites.

Material	Thermal Conductivity (W·m^−1^·K^−1^)	Bending Strength (MPa)
Uncoated diamond/Al	726	252 ± 9
Ti-coated diamond/Al	650	217 ± 14
CNT-modified Ti-coated diamond/Al	577	275 ± 6

## Data Availability

Data are contained within the article.
